# Assessing differences in healthcare access by HIV status to inform cervical cancer and HIV screening in rural Uganda

**DOI:** 10.1371/journal.pgph.0003918

**Published:** 2025-07-21

**Authors:** Mia Sheehan, Hallie Dau, Maryam AboMoslim, Priscilla Naguti, Nelly Mwandacha, Amy Booth, Candice Ruck, Laurie Smith, Jackson Orem, Gina Ogilvie, Carolyn Nakisige

**Affiliations:** 1 Faculty of Health Sciences, Simon Fraser University, Burnaby, Canada; 2 Women’s Health Research Institute, Vancouver, Canada; 3 School of Population and Public Health, UBC, Vancouver, Canada; 4 Virginia Commonwealth University, Richmond, Virginia, United States of America; 5 Uganda Cancer Institute, Kampala, Uganda; 6 BC Cancer, Vancouver, Canada; 7 BC Centre for Disease Control, Vancouver, Canada; Asian University for Women, BANGLADESH

## Abstract

Uganda has one of the highest incidence rates of cervical cancer in the world. Although this impacts all women, women living with human immunodeficiency virus (HIV) experience an increased risk for developing cervical cancer. This study aims to compare how HIV-positive and HIV-negative women in a remote sub-county in Uganda access health services to inform consideration of potential HIV and HPV-based cervical cancer screening integration at the community level. Women were recruited for this cross-sectional study door-to-door by village health teams if they had no prior screening or treatment of cervical cancer, no previous hysterectomy, were 30–49 years old residents of the South Busoga District Reserve, and could provide verbal informed consent. Participants completed a baseline survey, which included questions on HIV status, demographics, prior health history, past healthcare access and services recieved. The data was analyzed using bivariate descriptive statistics. Among the 1437 participants included in the analysis, 8.8% were HIV-positive. The majority of the respondents were between 30–34 years of age, were married, had received primary education or higher, and were farmers. The majority of women in both groups had accessed outreach visits (HIV-positive = 89.0%, HIV-negative = 85.8%) and health centres (HIV-positive = 96.1%, HIV-negative = 80.2%). The most commonly received services among both groups of women at outreach visits and health centres were immunization and antenatal care, respectively. Our study demonstrated that there were no significant differences in healthcare access between HIV-positive and HIV-negative women in rural Uganda. Additionally, the high usage of healthcare services by women living with HIV suggests that the integration of cervical cancer and HIV screening may facilitate early detection and prevention of cervical cancer among this population. This can reduce the burden of disease in Uganda and further contribute to the World Health Organization’s initiative to eradicate cervical cancer.

## Introduction

Cervical cancer is the most common cancer among women in low- and middle-income countries (LMICs) [1,2]. Despite cervical cancer being almost entirely preventable and treatable, LMICs face a significant burden of disease as prevention efforts such as vaccination and screening services are often limited [3,4]. Exacerbating the disproportionate burden of cervical cancer in LMICs, women are often diagnosed at advanced stages and are unable to receive effective treatment due to limited-service accessibility and availability [5]. Uganda has one of the highest incidence rates of cervical cancer in the world, with 56.2 reported cases per 100,000 women [3]. Despite the high burden of cervical cancer, less than 10% of Ugandan women have been screened [5], highlighting the urgent need for an accessible screening program across the country.

Healthcare access in rural areas is often hindered by logistical and health system barriers [6] such as long distances to health centres, inadequate resources at health facilities, and limited access to health information [7]. While improved screening is critical to achieve the World Health Organization’s global strategy to eliminate cervical cancer [8], it is important to understand the attendance to and utilization of health services in rural Uganda that are available [3]. This includes assessing attendance to healthcare and determining whether service use varies among women with higher risk for cervical cancer to inform an effective elimination strategy.

Women living with human immunodeficiency virus (HIV) are six times more likely to develop invasive cervical cancer compared to women without HIV infections, making cervical cancer prevention among this population a key priority [1,9,10]. Additionally, women living with HIV face many barriers to accessing health services such as financial burdens [11], limited awareness and availability of specialized care [11], and social stigma [11,12]. Ensuring access to cervical cancer screening for HIV-positive women may contribute to reducing HIV-attributable cervical cancer cases, and further alleviate the heightened burden of disease in Uganda [13]. To effectively facilitate cervical cancer prevention amongst this population, the integration of HIV and HPV-based cervical cancer screening may be beneficial. Sarah Maria et al. found that less than half of HIV-positive women in urban Uganda had ever been screened for cervical cancer [14], a number that is likely to be lower in rural areas. Integration could ultimately reduce barriers, specifically among women living in rural Uganda who experience additional challenges to accessing healthcare [7]. Additionally, literature from LMICs demonstrates that integration can improve the utilization of both HIV care and cervical cancer screening services, alleviate stigma, and enhance the overall quality of care received by patients, highlighting the benefits of this integration regardless of HIV status [15].

The potential for successful and effective integration of HIV and HPV-based cervical cancer screening services in Uganda relies on multiple factors, including understanding how women with HIV currently access health services compared to women without HIV. However, there is limited research on this topic, particularly in rural areas. The objective of this study is to compare how HIV-positive and HIV-negative women in a remote sub-county in Uganda access available health services to inform consideration of potential HIV and HPV-based cervical cancer screening integration.

## Materials and methods

### Design, setting, and study population

This cross-sectional study is part of a pragmatic cluster-randomized trial in Malongo, a rural and remote sub-county within the Mayuge district of Uganda focused on HPV-based self-collection for cervical cancer screening. The survey data used for this study is part of a baseline survey given to participants at enrollment in the pragmatic trial to assess knowledge and attitudes about cervical cancer and screening. Women were recruited door-to-door by village health teams (VHTs) between January 23 to August 24, 2023 from eleven villages in the South Busoga Forest Reserve in Malongo with the most limited access to healthcare. Door-to-door recruitment was used to ensure that we reach women who may have limited access to or no history utilizing health services [3]. Women were eligible for the study if they were aged 30–49 years old, reflecting the cervical cancer screening age of Uganda. Additionally, women were included in the study if they had not previously been screened for cervical cancer, did not have a previous hysterectomy, and could provide informed verbal consent. VHTs collected verbal consent and administered the survey on Research Electronic Data Capture (REDCap) software [16,17] using electronic tablets to ensure secure data collection. The consent form and all questions were read aloud in Lusoga or English, based on the language preference of the participant, to accommodate for the low literatcy rate among women in the region.

### Survey

The survey tool used in this study is informed by the Improving Data for Decision Making in Global Cervical Cancer Programs Toolkit-Part 2 (IDCCP) [18]. The survey included questions on participant demographics, medical history, knowledge about cervical cancer, and perceived barriers to screening.

To determine HIV status, women were asked if they had ever tested positive for HIV. Women had the option to respond with either yes, no, or prefer not to say. Women who answered yes (HIV-positive) or no (HIV-negative) were included in the study.

Several variables were used to assess access to health services. First, we asked women whether they had attended either a healthcare outreach visit or visited a health centre. Healthcare outreach visits included organized community health events or other instances where health services were available at the community level. Health centres refer to established clinics or hospitals that deliver healthcare services to patients. Response options for both variables were yes, no, and do not know. If a woman indicated do not know, it was recoded as no. Women were asked what services they received at the outreach visit and health centre visit, if applicable. Additionally, women were asked about how they were recruited to a health outreach visit and how they traveled to health centers.

Secondly, several variables related to cervical cancer screening were included to assess the potential integration of HIV care and cervical cancer screening. These variables included: prior awareness of cervical cancer, the barriers to cervical cancer screening, and perceived importance of early cervical cancer detection.

In addition to the variables described above, the survey asked several demographic questions that were used to understand the characteristics of the women participating in the study. The following demographic variables were included in the analysis: age, education level, occupation, relationship status, partner education level, age at first pregnancy, and number of pregnancies. Survey language was also reported.

### Data analysis

Bivariate descriptive statistics were used to analyze the findings of the survey data collected in the study to compare access to healthcare among women living with and living without HIV. All missing values were included in the results. Chi-square tests were performed, except in cases of low frequency, where Fisher’s exact test was used to determine statistically significant variables [19]. P-values ≤ 0.05 were considered statistically significant. R Studio (R 4.3.0) was used to conduct the analysis [20].

### Inclusivity in global research

Additional information regarding the ethical, cultural, and scientific considerations specific to inclusivity in global research is included in the Supporting Information (S1 Checklist).

### Ethics statements

Ethics approval was obtained from the University of British Columbia Children’s and Women’s Research Ethics Board (H22-01634), the Uganda Cancer Institute Research Ethics Committee (UCI-2022–56), and the Uganda National Council for Science and Technology. All participants provided informed verbal consent for their inclusion in the study. This consent was documented in REDCap and witnessed by the VHTs.

## Results

Overall, 1437 participants completed the survey, of which 127 women were HIV-positive (8.8%). The majority of the respondents in both groups (HIV-negative and HIV-positive) were between 30–34 years of age (HIV-positive n = 47, 37.0%; HIV-negative n = 565, 43.1%; p = 0.143), had obtained primary education or more (HIV-positive n = 76, 59.8%; HIV-negative n = 900, 68.7%; p = 0.123), were married (HIV-positive n = 107, 84.3%; HIV-negative n = 1136, 86.7%; p = 0.865), and were farmers (HIV-positive n = 99, 78.0%; HIV-negative n = 1117, 85.3%; p = 0.06). Additional information on the demographic characteristics of the survey respondents is provided in Table 1.

**Table 1:** Demographics stratified by HIV status.

	Overall	HIV-positive	HIV-negative	p-value
	N = 1437	N = 127	N = 1310	
	*n (%)*	*n (%)*	*n (%)*	
**Survey Language**				< 0.001
English	38 (2.6)	14 (11.0)	24 (1.8)	
Lusoga	1399 (97.4)	113 (89.0)	1286 (98.2)	
**Age**				0.143
30-34	612 (42.6)	47 (37.0)	565 (43.1)	
35-39	319 (22.2)	35 (27.6)	284 (21.7)	
40-44	249 (17.3)	17 (13.4)	232 (17.7)	
45-49	257 (17.9)	28 (22.0)	229 (17.5)	
**Education**				0.123
No Education	451 (31.4)	50 (39.4)	401 (30.6)	
Primary Education or more	976 (67.9)	76 (59.8)	900 (68.7)	
* Missing*	*10 (0.7)*	*1 (0.8)*	*9 (0.7)*	
**Occupation**				0.060
Farmer	1216 (84.6)	99 (78.0)	1117 (85.3)	
Other	178 (12.4)	21 (16.5)	157 (12.0)	
* Missing*	*43 (3.0)*	*7 (5.5)*	*36 (2.7)*	
**Relationship Status**				0.865
Married/In a relationship	1243 (86.5)	107 (84.3)	1136 (86.7)	
Single	116 (8.1)	12 (9.4)	104 (7.9)	
Separated/Divorced/Widowed	77 (5.4)	8 (6.3)	69 (5.3)	
* Missing*	1 (0.1)	0 (0.0)	1 (0.1)	
**Partner Education**				0.813
No Education	189 (13.2)	17 (13.4)	172 (13.1)	
Primary Education or more	1037 (72.2)	89 (70.1)	948 (72.4)	
* Missing*	*211 (14.7)*	*21 (16.5)*	*190 (14.5)*	
**Age at first pregnancy**				0.025
< 16	1194 (83.1)	96 (75.6)	1098 (83.8)	
≥ 16	243 (16.9)	31 (24.4)	212 (16.2)	
**Number of pregnancies**				0.875
1-6	40 (2.8)	3 (2.4)	37 (2.8)	
7-12	256 (17.8)	21 (16.5)	235 (17.9)	
≥ 13	1141 (79.4)	103 (81.1)	1038 (79.2)	

When participants were asked about their history of attendance at healthcare outreach visits, the majority of women, regardless of their HIV status, responded yes to attending (HIV-positive n = 113, 89.0%; HIV-negative n = 1124, 85.8%; p = 0.596). Among the 1237 women who attended a health outreach visit, immunization was reported as the most commonly accessed service for both groups (HIV-positive n = 59, 46.5%; HIV-negative n = 551, 42.1%; p = 0.388), followed by antenatal care (HIV-positive n = 47, 37.0%; HIV-negative n = 517, 39.5%; p = 0.655). When asked about how they were informed of health care outreach visits, the vast majority in both groups indicated VHT (HIV-positive n = 102, 80.3%; HIV-negative n = 1051, 80.2%; p = 0.036).

More women attended a health centre than healthcare outreach visits (HIV-positive n = 122, 96.1%; HIV-negative n = 1267, 96.7%; p = 0.289). Among the 1389 women who attended a health center, antenatal care was reported as the most commonly accessed service for both groups (HIV-positive n = 79, 62.2%; HIV-negative n = 839, 64.0%; p = 0.752) followed by HIV services for HIV-positive women (HIV-positive n = 41, 32.3%; p < 0.001) and family planning for HIV-negative women (HIV-negative n = 311, 23.7%; p = 0.749) (Table 2). The respondents in both groups reported relying mainly on walking as their primary means of transportation to reach health centres (HIV-positive n = 84, 66.1%; HIV-negative n = 839, 64.0%; p = 0.373).

**Table 2:** Results of survey on attendance to healthcare services stratified by HIV status.

	HIV+	Health Outreach	p-value		Health Centre	p-value
		HIV-		HIV+	HIV-	
	N = 127	N = 1310		N = 127	N = 1310	
	*n (%)*	*n (%)*		*n (%)*	*n (%)*	
**Attendance**	113 (89.0)	1124 (85.8)	0.596	122 (96.1)	1267 (96.7)	0.239
**Services Accessed**						
Antenatal Care	47 (37.0)	517 (39.5)	0.655	79 (62.2)	839 (64.0)	0.752
HIV	40 (31.5)	116 (8.9)	<0.001	41 (32.3)	147 (11.2)	<0.001
Family Planning	30 (23.6)	315 (24.0)	1.000	28 (22.0)	311 (23.7)	0.749
Immunizations	59 (46.5)	551 (42.1)	0.388	38 (29.9)	308 (23.5)	0.132

**Fig 1:**
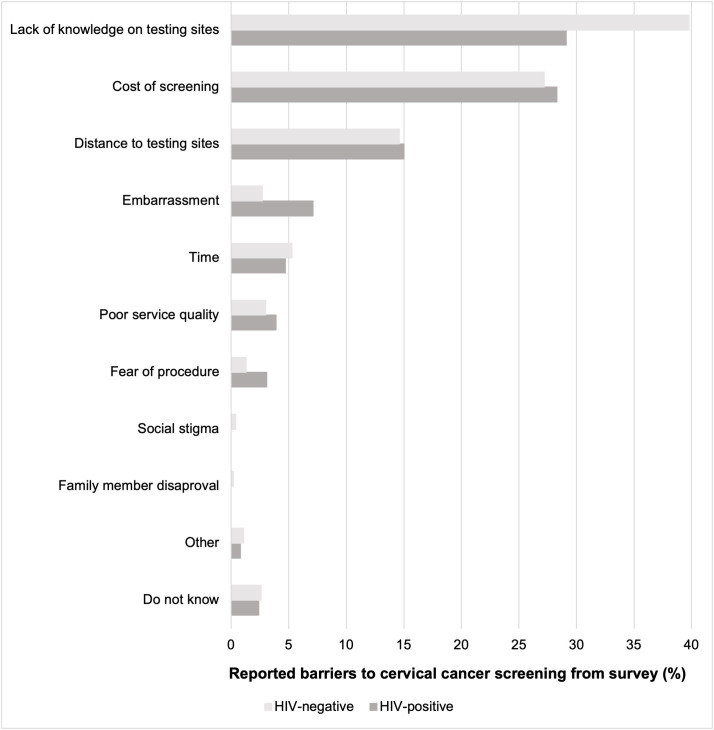
Common barriers to cervical cancer screening stratified by HIV status.

The majority of women across both groups answered that they had prior awareness of cervical cancer (HIV-positive n = 103, 81.1%; HIV-negative n = 1137, 86.8%; p = 0.321). Furthermore, the majority believed that early detection was important (HIV-positive n = 116, 91.3%; HIV-negative n = 1185, 90.6%; p = 0.01). When asked about the barriers to screening (Fig 1), the most common response among both groups was not knowing where to get tested (HIV-positive n = 37, 29.1%; HIV-negative n = 522, 39.8%) followed by the cost (HIV-positive n = 36, 28.3%; HIV-negative n = 356, 27.2%), and prohibitive distance to the clinic (HIV-positive n = 19, 15.0%; HIV-negative n = 191, 14.6%) (p = 0.023).

## Discussion

This study aimed to assess the differences in healthcare access by HIV status. We found that the majority of women in both HIV-positive and HIV-negative groups attended health care outreach visits and health centres and utilized similar services at both.

The majority of HIV-positive and HIV-negative women had prior awareness of cervical cancer and understood the importance of early cervical cancer detection. The lack of cervical cancer knowledge is likely not a barrier to accessing screening among never-screened women in Malongo. Research conducted in Zimbabwe [21], Ghana [22], and Western Uganda [23] on cervical cancer knowledge and attitudes of women found that women generally had good levels of understanding of HPV, cervical cancer, and screening [21–23]. However, findings demonstrated that knowledge and information on cervical cancer did not lead to behaviour change for screening, as represented by the low rates of screening among the women in these countries [21,23]. Additionally, there was no significant difference in knowledge between HIV-positive and HIV-negative women [22]. In Uganda, cervical cancer screening rates are less than 10% [5], despite the findings of our study indicating widespread awareness of cervical cancer in women, regardless of HIV status. There is a need for programming to bridge the gap between knowledge and behaviour change to ensure that awareness is translated into increased screening to alleviate the burden of disease among women in Uganda [24].

The majority of women in both groups who attended health outreach visits were informed about these visits by VHTs. Current literature demonstrates the critical role that VHTs play in disseminating health information and facilitating access to health services in Uganda [25]. As a primary point of contact for health and social services, VHTs assist community members in understanding available healthcare services and options and mobilize individuals in the community to actively participate in accessible health programs [25,26]. Their role in the healthcare of Uganda can help overcome barriers to participation and encourage community members to access services available to them. The findings from our study along with the evidence provided in the literature suggest that VHTs could play a critical role in raising awareness of HPV-based cervical cancer screening and available screening options in both HIV-positive and negative populations. Moreover, VHTs may contribute to the promotion and establishment of the successful integration of HIV and HPV-based cervical cancer screening services in rural communities.

Our study indicated that all women had a history of pregnancy and that a high proportion of HIV-positive and HIV-negative women had received antenatal services, similar to findings that have shown a high rate of attendance to antenatal care among pregnant women in Uganda [27]. In addition to the notable overlap of the age of women attending antenatal care and the age of women recommended to be screened for cervical cancer [28], pregnancy is a period where women experience heightened medical supervision and prioritize their own health conditions [29]. Furthermore, women attending antenatal care show a strong commitment to adhere to follow-up visits and care instructions throughout their pregnancy [30]. Therefore, the antenatal period may provide an opportunity for increased uptake of HPV-based cervical cancer screening, both in HIV-negative and HIV-positive women in Uganda [29].

Given that HIV-positive women are at a higher risk for developing cervical cancer, it is critical to prioritize cervical cancer prevention amongst this population [1,9,10]. Investigating the differences in healthcare service utilization between HIV-positive and HIV-negative women allows for a better understanding of additional barriers that women living with HIV may be facing in accessing cervical cancer prevention services. This study highlighted that there were no significant differences in health service usage between HIV-positive and HIV-negative women. This demonstrates the ways in which women living with HIV are generally similarly engaged with the healthcare system in rural Uganda compared to HIV-negative women. This may further suggest that health inequities faced by women living with HIV are not being exacerbated by barriers to accessing and utilizing available health services, suggesting that lack of access does not fully explain the low uptake rates of cervical cancer screening reported among HIV-positive women [14]. These findings can inform additional investigation into the barriers that prevent the uptake of cervical cancer screening in this population., as well as the development of a comprehensive HPV-based cervical cancer screening in Uganda that effectively targets women regardless of their HIV status. Further, this may lead to increased access to reproductive services among both HIV-positive and negative women in rural and remote communities, allowing for expanded health access for all. Integrated, concurrent screening for HIV and HPV may be more effective in the early detection and treatment of pre-cancerous lesions among women living with HIV, reducing the burden of disease that these women face [31].

The findings of this study are strengthened by the experience and the expertise of the study team. Additionally, study recruitment and survey administration were also conducted door-to-door, meaning women were involved in the study from the safety and comfort of their own homes. Despite the strengths of this study, some limitations exist. As the prevalence of HIV was much higher in the women who were surveyed in comparison to the population data of women living in Uganda, the results of this study may not be generalizable [32]. This difference in HIV prevalence may be due to self-reporting of HIV status, which could impact the results of the study. Similarly, the collected survey data was based on self-reported information, which may be subject to response biases that influence the results of the study [33]. As women in the study responded to the survey questions to VHTs verbally, this may have resulted in social desirability bias [34].

The results from our study demonstrate that women in remote Uganda had high attendance to health services offered at both outreach visits and health centres, with no significant differences between HIV-positive and HIV-negative women. Further research is needed on how to effectively integrate HIV and HPV-based cervical cancer screening programs into rural health systems at the community level.

## Supporting information

S1 Checklist Inclusivity in global research. (DOCX)
